# Psychometric testing of the Korean version of the Wijma Delivery Expectancy Questionnaire: a methodological study

**DOI:** 10.4069/whn.2025.12.02

**Published:** 2025-12-31

**Authors:** Seyeon Park, Sukhee Ahn, Hyunjin Cho

**Affiliations:** College of Nursing, Chungnam National University, Daejeon, Korea

**Keywords:** Childbirth, Fear, Surveys, Validation study

## Abstract

**Purpose:**

Childbirth-related fears contribute to both physical and psychological challenges in women, including complicated deliveries and postpartum depression. In South Korea, these fears have primarily been assessed using generalized anxiety measurement tools, which have limited accuracy because they do not specifically capture fear related to childbirth. This study aimed to translate the Wijma Delivery Expectancy Questionnaire (W-DEQ) into Korean and to evaluate its reliability and validity.

**Methods:**

A cross-sectional survey design was employed. The Korean version of the W-DEQ was developed using a forward-backward translation process. Data obtained from face-to-face surveys conducted with 200 women were analyzed to evaluate the psychometric properties of the instrument, including its validity and reliability.

**Results:**

The mean age of the participants was 33 years; 32.5% were aged 35 years or older, and 61% were nulliparous expectant mothers. Confirmatory factor analysis identified the best-fitting model and supported construct validity. The Korean W-DEQ consists of 26 items rated on a 6-point scale and encompasses four factors: fear, lack of expectation, isolation, and perceived risk. All factor loadings were ≥0.4, and all item–total correlations exceeded 0.2, indicating acceptable reliability. Convergent and discriminant validity testing supported construct validity. Significant correlations between the Korean W-DEQ and measures of depression and childbirth confidence confirmed its concurrent validity. Nulliparous women scored significantly higher on perceived risk of baby injury than did parous women.

**Conclusion:**

The Korean W-DEQ demonstrated satisfactory reliability and validity, confirming its usefulness for assessing maternal fear of childbirth in both nulliparous and multiparous women.

## Introduction

Pregnancy and childbirth are significant transitional events in a woman’s life. During these periods, women often experience ambivalence, simultaneously reporting positive emotions such as pleasure and anticipation and negative emotions such as anxiety and fear [1]. Fear of childbirth (FOC) refers specifically to a negative cognitive evaluation of the childbirth experience and is associated with feelings of fear and anxiety [2]. Women frequently report fears related to childbirth, including concerns about pain, anxiety regarding potential complications, and worries about their own survival as well as their baby’s well-being. In addition, women may experience a perceived loss of control and feelings of social isolation during this period [1]. Extreme FOC has been reported in 6%–15% of pregnant women in Europe, 2%–24% in North America, and 26%–29% in Australia [1,3-6]. In Asia, the prevalence of severe fear has been reported as 26.5% among Korean women [7], 41% among Asian Turks [8], and 60%–75% among Iranian women [9]. By contrast, Chinese women generally report moderate levels of childbirth-related fear [10].

A recent meta-analysis demonstrated that FOC is influenced by a range of factors that, in turn, affect both childbirth outcomes and postpartum health [11]. These factors include age, race, parity, educational level, and economic status. In addition, multiple studies have consistently reported associations between increased FOC and psychological and social variables, including stress, anxiety, depression, insufficient social support, and low self-efficacy related to childbirth during pregnancy [8,11-13].

More intense FOC is associated with an increased likelihood of cesarean section [1,14]. This fear is also linked to a higher risk of pregnancy-related complications, including difficult labor, delayed delivery, and fetal distress. Consequently, the use of epidural anesthesia and the incidence of cesarean delivery are increased among women with elevated FOC [8,11,15]. Expectant mothers who experience substantial childbirth-related fear may also encounter difficulties in bonding with their infants, report reduced self-esteem, and show an increased risk of postpartum depression [16,17]. Furthermore, these women are at heightened risk of developing post-traumatic stress disorder following childbirth [11,17,18].

Previous studies have reported inconsistent findings regarding whether FOC differs according to parity. While several studies have found no significant differences based on parity [1,7,19,20], others have reported that primiparous women experience greater FOC than women who have previously given birth [21,22]. The underlying reasons for fear also appear to differ by parity. First-time mothers often report uncertainty related to the delivery process due to a lack of personal experience. In contrast, multiparous women may experience heightened fear stemming from previous negative childbirth experiences, such as emergencies or operative deliveries [11]. Therefore, early identification of pregnant women with elevated FOC is essential. In addition, the provision of appropriate psychosocial interventions during pregnancy and the postpartum period, along with the development of individualized delivery plans, is critical for preventing postpartum depression and promoting maternal–infant bonding.

In Korea, assessment of FOC among pregnant women has traditionally relied on general anxiety measurement tools [23,24]. However, these instruments may not adequately capture the specific characteristics or severity of childbirth-related fear, as anxiety during pregnancy is known to be closely associated with, but distinct from, FOC [25-27]. To address this limitation, the Wijma Delivery Expectancy Questionnaire (W-DEQ), developed by Wijma et al. [27], has been widely used to assess delivery-related fear [1]. The original 33-item instrument was developed and validated among pregnant Swedish women [27] and includes multiple subdomains that have been examined across different cultural contexts. These subdomains encompass fear, negative sentiments or evaluations, lack of positive anticipation, feelings of isolation, low self-efficacy, diminished confidence, perceived risks, and hope for the baby. Validation studies conducted in different countries have yielded varying factor structures, including a 25-item, six-factor version in Australia [26] and the original 33-item, four-factor structure identified in Australia [28], the United Kingdom [29], Japan [30], and Sweden [31]. These variations suggest that FOC is shaped by personal experiences, delivery history, and culturally specific perceptions of pregnancy and childbirth. Accordingly, the psychometric validity of the W-DEQ should be evaluated within each cultural context rather than being directly adapted without validation. Despite the widespread international use of the W-DEQ to assess women’s FOC, a psychometrically validated Korean version has not yet been established. Therefore, this study aimed to evaluate the reliability and validity of the Korean version of the W-DEQ (KW-DEQ).

The null hypothesis of this study was that the KW-DEQ would not demonstrate adequate reliability or validity, nor would it show significant associations with related variables such as depression and childbirth confidence.

## Methods

**Ethics statement:** Ethics approval was granted by the Institutional Review Board of the Chungnam National University (No. 201904-SB-044-01). Participation in the study was voluntary, and all participants provided written informed consent prior to enrollment.

### Study design and participants

This methodological study was conducted to translate the W-DEQ into Korean and to evaluate its validity and reliability. It was a secondary analysis of the original dataset that explored factors influencing FOC among pregnant women [7]. A cross-sectional design was employed, and 200 pregnant women were recruited based on the following inclusion criteria: gestational age of at least 28 weeks, age of 20 years or older, singleton pregnancy, expectation of vaginal delivery, and sufficient proficiency in reading Korean.

The sample size was determined according to the guidelines proposed by Costello and Osborne [32], who recommend a minimum sample size of 5 to 10 participants per item for exploratory factor analysis to ensure adequate construct validity. As the original W-DEQ includes 33 items, a minimum sample size ranging from 165 to 330 participants was considered necessary.

### Translation procedure

The translation process for the FOC measurement tool followed the double-translation method proposed by Lenz [33]. Initially, two independent translators translated the original English version of the W-DEQ into Korean while remaining unaware of the study’s purpose. A nursing professor subsequently compared the two translated versions and facilitated consensus. A nurse with experience in obstetric care then reviewed the translated items to assess cultural appropriateness within the Korean context. Following this review, a bilingual individual conducted a back-translation into English. A PhD-level nursing researcher from the United States with childbirth experience compared the original and back-translated versions to confirm that the meaning of each item was preserved. Finally, the researcher verified that the terminology used in the finalized Korean version was consistent with the Standard Korean Language Dictionary published by the National Institute of the Korean Language.

During the translation process, the term “abandoned” in item 15 was translated as “neglected” to better reflect cultural nuances in Korea, where the presence of husbands during childbirth is common. The original term was considered potentially provocative in this context, making “neglected” a more culturally appropriate alternative. In addition, the term “panic” in item 19 was found to be insufficiently clear within the context of childbirth. Accordingly, it was revised to “confused,” a term that is more familiar and meaningful in Korean usage. This comprehensive translation process was designed to incorporate cultural considerations related to childbirth in Korea while maintaining the reliability and validity of the original instrument.

### Measurements

#### Fear of childbirth: Wijma Delivery Expectancy Questionnaire version

The W-DEQ, originally developed by Wijma et al. [27], was used with permission from the original authors. This instrument consists of 33 items rated on a 6-point Likert scale (0–5) and is designed to assess a woman’s cognitive and emotional expectations regarding childbirth. The five overarching questions address the following domains: (1) overall expectations regarding labor and delivery, (2) general emotional responses during labor and childbirth, (3) anticipated feelings throughout the labor and birth process, (4) expectations during the most intense phase of labor, and (5) anticipated feelings at the moment of birth.

Negatively worded items (items 2, 3, 6, 7, 8, 11, 12, 15, 19, 20, 24, 25, 27, and 31) were reverse-scored prior to analysis. Total scores on the original W-DEQ range from 0 to 165, with higher scores indicating greater FOC. A score of 85 or higher is generally considered indicative of clinically severe fear. At the time of its development, the instrument demonstrated strong internal consistency, with a reported Cronbach’s α of 0.90. Previous validation studies have consistently identified four underlying factors: fear, lack of positivity, isolation, and perceived risk [28-31].

#### Childbirth confidence

Childbirth confidence was measured using the Childbirth Confidence Scale developed by Lee [34]. This scale consists of 15 items rated on a 4-point Likert scale (1–4), yielding a total possible score ranging from 15 to 60. Higher scores reflect greater confidence in childbirth. The instrument demonstrated good reliability, with a Cronbach’s α of 0.87 in the original validation study and 0.86 in the present study.

#### Prenatal depression

Depression was assessed using the Korean version of the Edinburgh Postnatal Depression Scale (EPDS) [35], originally developed by Cox et al. [36]. The Korean EPDS includes 10 items rated on a 4-point scale (0–3), with total scores ranging from 0 to 30. Higher scores indicate greater severity of depressive symptoms. The original and Korean versions of the scale reported Cronbach’s α values of 0.87 and 0.86, respectively. In the current study, internal consistency remained high, with a Cronbach’s α of 0.86.

### Data collection

Following Institutional Review Board approval, 230 pregnant women who met the inclusion criteria were recruited from a women’s health hospital. Participants who agreed to take part in the study completed a self-administered questionnaire. Data collection occurred between September 2019 and February 1, 2020. Information on participants’ demographic and obstetric characteristics, including age, parity, and pregnancy planning status, was collected. After excluding incomplete responses, data from 200 participants were included in the final analysis.

### Data analysis

Data were analyzed using SPSS version 29.0 and AMOS version 29.0 (IBM Corp., Armonk, NY, USA), with the level of statistical significance set at 0.05. Participants’ general characteristics were summarized using frequencies and descriptive statistics. The construct validity of the KW-DEQ was evaluated through confirmatory factor analysis, with factor loadings of 0.40 or higher considered acceptable. Construct validity refers to the degree of correspondence between a theoretical construct and its observed indicators; accordingly, both convergent and discriminant validity were examined. Convergent validity was established by confirming construct reliability values greater than 0.70 or average variance extracted values exceeding 0.50. Discriminant validity was assessed by verifying that the inter-factor correlation coefficient ±(2×standard error) did not include the value of one [37].

Concurrent validity was examined by assessing Pearson correlation coefficients between the total and subscale scores of the KW-DEQ and measures of childbirth confidence and depression. Known-group validity was evaluated by comparing KW-DEQ scores according to parity using the independent t-test. Finally, the internal consistency reliability of the finalized instrument was assessed by calculating Cronbach’s α.

## Results

### Sample characteristics

The mean age of the participants was 33.12 years (standard deviation [SD], 5.40), with 135 participants (67.5%) aged under 35 years. Most participants had attained a college degree or higher (92.5%), and more than half were classified as belonging to the low-to-middle socioeconomic group (57.0%). The mean gestational age was 34.07 weeks (SD, 3.62), with 61.0% of participants being first-time mothers and 67.5% reporting a planned pregnancy (Table 1).

### Korean version of the Wijma Delivery Expectancy Questionnaire: construct validity test

#### Confirmatory factor analysis

Confirmatory factor analysis using maximum likelihood estimation was conducted to test the four-factor structure previously identified in a Japanese study [30], which was selected due to its shared Asian cultural context of childbirth. The four factors included fear, lack of expectation, isolation, and perceived risk. Comparison of model fit indices across three models showed that the first model, which included all 33 items, demonstrated poor fit. The second model, which excluded two items with negative factor loadings, did not yield meaningful improvement in model fit. In contrast, after removing seven items with factor loadings below 0.40 and adding correlated error paths based on modification indices, the fit of the third model improved substantially. This final model comprised 26 items. The fit indices for the final model were as follows: chi-square minimum, 2.91; root mean square error of approximation (RMSEA), 0.09; comparative fit index (CFI), 0.78; and Tucker–Lewis index (TLI), 0.75. Correlation coefficients among the four factors were all statistically significant and ranged from 0.13 to 0.75. Factor loadings ranged from 0.42 to 0.75 for fear, 0.44 to 0.85 for lack of expectation, 0.45 to 0.82 for isolation, and 0.87 to 0.96 for perceived risk, with all loadings reaching statistical significance (*p*<.001) (Tables 2, 3).

#### Convergent and discriminant validity

Construct validity was evaluated through assessment of both convergent and discriminant validity. Convergent validity was supported by average variance extracted values exceeding 0.50 for all four factors, along with construct reliability values greater than 0.70 (Table 3). Discriminant validity was confirmed by demonstrating that the inter-factor correlation coefficients, calculated as ±(2×standard error), ranged from 0.25 to 0.72 and did not include the value of 1.

#### Criterion validity: relationship between Korean version of the Wijma Delivery Expectancy Questionnaire scores and anxiety

Criterion validity was assessed by examining correlations between the Korean W-DEQ and measures of childbirth confidence and depression. Significant positive correlations were identified between depression and both the total score and three of the four factor scores of the Korean W-DEQ, with the exception of the fear factor (r=0.14–0.24). In contrast, both the total score and all four factor scores of the Korean W-DEQ were significantly negatively correlated with childbirth confidence (r=−0.20 to −0.45) (Table 4).

Nulliparous mothers had a mean total KW-DEQ score of 57.75±17.40, which was comparable to the mean total score of 59.359±17.74 observed among parous mothers. Across the four factors, no significant group differences were found for the subscales of fear, lack of expectation, or isolation. However, for the subscale of perceived risk factor, parous mothers scored significantly higher (3.43±3.00) than nulliparous mothers (1.80±2.42) (t = −4.22, *p*<.001) (Table 5).

### Reliability analysis

The overall internal consistency reliability of the KW-DEQ was high, with a Cronbach’s α of 0.90. Cronbach’s α values for the individual factors ranged from 0.77 to 0.91, including fear (0.77), lack of expectation (0.77), isolation (0.79), and perceived risk (0.91). These findings indicate that the instrument demonstrated acceptable to excellent reliability. Correlation coefficients between the total KW-DEQ score and its sub-factor scores ranged from 0.47 to 0.93 (*p*<.001), while correlations among the sub-factors ranged from 0.12 to 0.59 (Table 4).

## Discussion

This study was conducted to translate the widely used W-DEQ into Korean and to evaluate its reliability and validity. The KW-DEQ, consisting of four factors and 26 items as confirmed in this study, offers the advantage of capturing the nuanced psychological aspects of pregnant women’s FOC.

Since the W-DEQ was initially developed as a single-factor model in 1998, numerous subsequent studies have identified multiple underlying factors [25,28-31], raising questions about the necessity and usefulness of simpler sub-factor structures. In the present study, we conducted a series of confirmatory factor analyses based on a four-factor structure derived from a Japanese study [30], which was selected due to its similar cultural context for childbirth. We found that the 26-item model demonstrated the best overall fit. The four-factor KW-DEQ model showed superior fit compared with the original 33-item single-factor model [27] and other previously proposed four-factor models [28-31]. The KW-DEQ was categorized into four factors—fear, lack of expectations, isolation, and perceived risk—which align with factors identified in earlier W-DEQ validation studies [28-31]. The KW-DEQ effectively captures a broad range of childbirth-related fears, including perceptions of risk during childbirth, feelings of isolation, and a lack of positive expectations regarding the labor and delivery process. These findings are consistent with previous research showing that pregnant women in Australia hold diverse expectations regarding childbirth, viewing it both as a natural event associated with satisfaction and as a potentially negative experience that evokes fear [38]. In contrast, Korean pregnant women tend to report greater uncertainty about childbirth, lower confidence in natural birth, and persistent FOC from early pregnancy through delivery [39]. One possible explanation for this discrepancy is the degree of medical intervention during childbirth. In Korea, childbirth is highly medicalized, with high rates of cesarean section and widespread use of epidural anesthesia. Within such a medicalized system, childbirth may be perceived primarily as a medical procedure rather than a natural physiological process, which may contribute to reduced confidence in vaginal delivery and heightened FOC [40]. Given these sociocultural characteristics, it is essential to use an assessment tool that accurately reflects the childbirth experiences of Korean women. Accordingly, the KW-DEQ was developed to capture these contextual differences rather than directly adopting the original W-DEQ without cultural adaptation.

In this study, the reliability and validity of the KW-DEQ were evaluated, and the findings indicated acceptable internal consistency for both the total score and the individual sub-factors. In addition, the KW-DEQ demonstrated a moderately negative correlation with childbirth confidence and a weak positive correlation with depression, supporting its criterion validity. These results are consistent with previous studies indicating that lower childbirth confidence and higher levels of prenatal depression are associated with increased FOC [6,7].

Total FOC scores and three of the four factors were similar between nulliparous women and those with previous childbirth experience, suggesting that FOC may be experienced by pregnant women regardless of parity. Previous research examining parity-related differences in childbirth fear has produced inconsistent findings [7,8,21,22]. Several studies have reported that nulliparous women generally experience higher levels of childbirth fear than multiparous women; for example, a study involving 200 pregnant women found significantly higher W-DEQ scores among nulliparous participants [21,22]. In contrast, in the present study, parous women reported significantly higher scores for perceived risk of infant injury compared with nulliparous women. This finding suggests that prior childbirth experience does not necessarily attenuate fear and may, in some cases, heighten concern regarding adverse outcomes. Supporting this interpretation, a large Finnish study reported a higher prevalence of severe childbirth fear among multiparous women compared with nulliparous women (4.5% vs. 2.5%) [41]. This paradox has been attributed to the impact of negative or traumatic birth experiences, as women with prior adverse or emergency deliveries tend to anticipate greater complications in subsequent pregnancies [42].

These findings are consistent with evidence suggesting that first-time mothers experience significantly greater anxiety and depression related to potential risks to their babies during childbirth [1,2]. Nulliparous women may lack detailed knowledge of the delivery process, leading to overestimation of physical risks and complications associated with labor and delivery. Encouraging first-time mothers to participate in childbirth education programs may help reduce anxiety, depression, and FOC by improving knowledge, providing emotional support, and strengthening confidence [43]. In contrast, women with previous childbirth experience may assess delivery-related risks more realistically and report a greater sense of control based on their prior experiences [44]. Therefore, early identification of pregnant women with elevated levels of childbirth fear is critical. Implementing psychological interventions tailored to individual fear levels and prior childbirth experiences may improve expectations and enhance confidence in managing the childbirth process.

This study has several limitations. First, the sample size was smaller than the recommended sample size-to-item ratio for psychometric validation, which may limit the robustness of the findings and warrants cautious interpretation. Second, although the four-factor structure demonstrated acceptable construct validity, certain model fit indices showed limitations. Specifically, the CFI and TLI values were lower than ideal cutoff thresholds, and the RMSEA value was slightly higher than desired. These results suggest that the model may not fully capture the complexity of childbirth fear among Korean women, indicating a need for further refinement of scale items or exploration of alternative model structures in future studies. Nevertheless, multiple analytic approaches—including convergent and discriminant validity testing, reliability analysis, criterion validity, and known-group comparisons—provided consistent evidence supporting the psychometric soundness of the KW-DEQ. Given its acceptable reliability and validity across diverse analyses, the instrument is expected to contribute meaningfully to future research and clinical assessment of childbirth fear among Korean women. Future studies should include larger sample sizes to more accurately estimate levels of childbirth fear, identify high-risk groups, and examine associated factors. Additionally, further refinement of the instrument to better reflect sociocultural dimensions of childbirth fear is warranted. Finally, feasibility validation of a revised FOC instrument (version 2) is recommended to examine alignment between women’s prenatal expectations and postnatal experiences.

In conclusion, the KW-DEQ is a reliable and valid instrument that effectively captures the diverse fears experienced by pregnant women during childbirth, including feelings of isolation, expectations regarding delivery, fear, perceived self-control, and perceived risk. Furthermore, the KW-DEQ may serve as a valuable tool for developing and implementing interventions aimed at reducing childbirth-related fear. Its application may enhance targeted prenatal and postpartum education by helping align women’s expectations with their actual childbirth experiences. In addition, nurses should provide counseling and informational support tailored to individual levels of childbirth fear, regardless of parity.

## Figures and Tables

**Table 1.** General characteristics of participants (N=200)

Variable	Categories	Mean±SD or n (%)
Age (year)		33.12±5.40
	<35	135 (67.5)
	≥35	65 (32.5)
Education level	High school	15 (7.5)
	College and above	185 (92.5)
Subjective economic status	Upper class	86 (43.0)
	Low/middle class	114 (57.0)
Gestational period (week)		34.07±3.62
	<36	149 (65.4)
	≥35	79 (34.6)
Parity	Nulliparous	122 (61.0)
	Parous	78 (39.0)
Planned pregnancy	Yes	135 (67.5)
	No	65 (32.5)

**Table 2.** Model fit statistics for Korean W-DEQ models (N=200)

Model No.	CMIN	*p*-value	CFI	TLI	RMSEA	Items excluded
1	3.62	<.001	.57	.54	.11	-
2	3.62	<.001	.62	.55	.11	Q4, 10
3	2.91	<.001	.78	.75	.09	Q4, 10, 9,21,28,30,31

W-DEQ, Wijma Delivery Expectancy Questionnaire CMIN: Chi-square value; CFI: comparative fit index; TLI: Tucker-Lewis index; RMSEA: root mean square error of approximation.

**Table 3.** Confirmatory factor analysis of the Korean W-DEQ (N=200)

No	Variable	Korean W-DEQ				
		Mean±SD	Fear	Lack of expectation	Isolation	Perceived risk
17	Relaxed	3.07±1.24	.75			
26	Allow my body to take control	2.76±1.19	.67			
22	Self-confidence	2.54±1.25.	.65			
6	Afraid (R)	2.98±1.18	.62			
5	Confident	3.05±1.33	.59			
16	Composed	2.66±1.26	.59			
19	Confused (R)	2.61±1.38	.58			
27	Lose control of myself (R)	2.24±1.15	.51			
2	Frightful (R)	3.10±1.29	.51			
12	Tense (R)	3.32±1.20	.50			
29	Natural	1.66±1.26	.49			
25	Behave badly (R)	1.59±1.13	.46			
24	Painful (R)	3.67±1.28	.45			
23	Trust	2.00±1.04	.42			
18	Happy	1.91±1.22		.85		
13	Glad	1.75±1.28		.69		
14	Proud	1.61±1.21		.59		
1	Fantastic	3.14±1.38		.44		
8	Weak (R)	2.05±1.24			.82	
7	Deserted (R)	2.62±1.19			.64	
20	Hopelessness (R)	1.33±1.21			.58	
11	Desolate (R)	1.54±1.29			.53	
3	Lonely (R)	1.72±1.37			.49	
15	Neglected (R)	3.07±1.24			.45	
32	Baby will die	1.18±1.52				.96
33	Baby will be injured	1.26±1.37				.87
Construct reliability (CR)			.86	.75	.75	.91
Average variance extracted			.32	.44	.39	.84
Cronbach’s alpha		.90	.77	.77	.79	.91

W-DEQ, Wijma Delivery Expectancy Questionnaire. (R) indicates a reverse-graded item.

**Table 4.** Association of the Korean W-DEQ with related variables (N=200)

Variable	r (*p*)				
	Total	Fear	Lack of expectation	Isolation	Perceived risk
Korean W-DEQ					
Fear	.93 (<.001)	1			
Lack of expectation	.64 (<.001)	.45 (<.001)	1		
Isolation	.78 (<.001)	.59 (<.001)	.44 (<.001)	1	
Perceived risk	.47 (<.001	.36 (<.001)	.12 (.079)	.25 (<.001)	1
Childbirth confidence	–.45 (<.001)	–.41 (<.001)	–.27 (<.001)	–.39 (<.001)	–.20 (.005)
Depression	.20 (.005)	.13 (.068)	.14 (.048)	.22 (.002)	.24 (<.001)

W-DEQ, Wijma Delivery Expectancy Questionnaire.

**Table 5.** Comparison of the Korean W-DEQ between nulliparous and parous groups (N=200)

Korean W-DEQ	Mean±SD			t (*p*)
	Total	Nulliparous (n=122)	Parous (n= 78)	
Fear	37.18±10.52	37.58±9.86	36.79±11.18	0.52 (.466)
Lack of expectation	8.43±3.86	8.32±4.12	8.55±3.60	–0.39 (.189)
Isolation	10.32±5.22	10.04±5.19	10.61±5.25	–0.75 (.823)
Perceived risk	2.25±2.71	1.80±2.42	3.43±3.00	–4.22 (<.001)
Total	58.57±17.57	57.75±17.40	59.39±17.74	–0.64 (.813)

W-DEQ, Wijma Delivery Expectancy Questionnaire.
